# Green Method Comparison and Optimization of Anthocyanin Recovery from “Sangiovese” Grape Pomace: A Critical Evaluation of the Design of Experiments Approach

**DOI:** 10.3390/molecules29112679

**Published:** 2024-06-05

**Authors:** Mariacaterina Lianza, Fabiana Antognoni

**Affiliations:** Department for Life Quality Studies, University of Bologna, Corso d’Augusto 237, 47921 Rimini, Italy; mariacaterina.lianz3@unibo.it

**Keywords:** anthocyanins, Design of Experiments, grape pomace, green extraction, MAE, MODDE, UAE, NaDES, response surface model

## Abstract

Grape pomace is the main by-product obtained from wine production that is still enriched in bioactive compounds. Within a framework of waste/by-product reuse through a sustainable approach, various green methods were utilized in this work to recover anthocyanins from the pomace resulting from “Sangiovese” grape vinification. Ultrasound- and Microwave-Assisted Extractions (UAE and MAE) were coupled with the use of green solvents, such as acidified water, an ethanol/water mixture, and Natural Deep Eutectic Solvents (NaDES), and their efficacy was compared with that of a conventional method based on a methanol/acidified water mixture. The Total Anthocyanin Index ranged from 36.9 to 75.2 mg/g DW for UAE, and from 54.4 to 99.6 mg/g DW for MAE, while resulting in 47.1 mg/g DW for conventional extraction. A Design of Experiments (DoE) approach was applied to MAE, the most efficient technique. Temperature, time, and the solid-to-liquid ratio were set as X variables, while malvidin-3-*O*-glucoside content and antioxidant activity were used as response variables, measured by High-Performance Liquid Chromatography with Diode Array Detection (HPLC-DAD) and 2,2-Diphenyl-1-picrylhydrazyl (DPPH) assay, respectively. The correlation between temperature and time and the antioxidant activity of the extract was positive, while it was found to be negative when considering malvidin-3-*O*-glucoside concentration as a response variable. Thus, the optimal conditions in temperature, time and solid-to-liquid ratio were different depending on the chosen variable. The results underline the importance of selecting an accurate response when using the response surface methodology approach.

## 1. Introduction

In recent decades, wastes and by-products derived from fruit and vegetable processing have been recognized as a valuable source of bioactive compounds, which can be used to produce added value-functional ingredients for foods, supplements, cosmetics, and pharmaceuticals, among others [1,2].

The exploitation of plant-derived wastes and by-products, and the development of more sustainable methods for extracting and utilizing functional compounds still present in these matrices represent important goals to be achieved to meet the recent priorities established by the European Union (EU). Despite recognizing the fundamental role of chemicals for human well-being, the EU Chemicals Strategy for Sustainability, adopted in 2020, sets out pragmatic actions to reduce the adverse impact of chemicals on the environmental and human health [3]. In this framework, the scientific and industrial communities all over the world are adopting novel and greener approaches for extracting bioactive compounds from natural sources. These include the substitution of the most used organic solvents, such as methanol and hexane, with less toxic and more eco-friendly ones. Besides more sustainable solvents, the green extraction of natural products also leads to the use of green techniques, which allow for a reduction in energy consumption, extraction time, unit operations, and volumes of solvents compared to the traditional techniques, meanwhile improving the yield and the quality of the obtained extract/product [4].

Ultrasound-Assisted Extraction (UAE) and Microwave-Assisted-Extraction (MAE) have been widely applied for extracting natural products, and very exhaustive reviews on these topics have been published, covering the rationale underlying their mechanisms of action and their efficiency [5,6,7]. Among the green solvents, Natural Deep Eutectic Solvents (NaDES) have recently gained a growing interest as a greener alternative to conventional solvents. They are usually obtained by the complexation of a quaternary ammonium salt with a metal salt or hydrogen bond donor (HBD) and offer several advantages, such as nonflammability, low vapor pressure, and a relatively wide liquid range [8]. Moreover, they are obtained by combining molecules largely occurring in living cells that might constitute a sort of “third liquid phase” particularly suitable for solubilizing, storing, or transporting water-insoluble compounds [9]. These characteristics have recently led them to be chosen as the solvent mixture of choice in a variety of scientific and technological areas [10,11], including the extraction processes of different classes of polar and non-polar bioactive products from plant matrices, such as coumarins [12], curcumins [13], phlorotannins [14], and anthocyanins [15]. 

Grape pomace is the main solid waste remaining from the winemaking process, which is composed of seeds, skin, small pieces of stalks, residues of pulp, and, in the case of red pomace, yeast cells [16]. Since it represents about 20–25% of the grape weight, the production of grape pomace can reach very high levels, especially in countries that are leading producers of wine, and this rises the problem of the proper disposal of this solid waste to reduce the environmental impact in terms of energy consumption, water pollution, and soil contamination. For these reasons, several studies have been recently carried out with the aim of reusing this residue as a source of energy and/or value-added products for obtaining economic and environmental benefits. For example, different chemical, physical and biological strategies have been applied to transform grape pomace into fertilizers [17], biofuels [18], biochar [19], and food additives and nutraceutical ingredients have been obtained by virtue of the antioxidant/antibacterial, and health-promoting activities of compounds still present in it, such as polyphenols, fibers, unsaturated fatty acids, and vitamins [20,21,22,23]. One of these valuable specialized metabolites in grape pomace is represented by anthocyanins, a class of water-soluble flavonoids possessing a wide array of biological properties. Apart from the well-known antioxidant capacity [24], many other health-beneficial effects have been demonstrated, ranging from antimicrobial [25] to the prevention of chronic and degenerative diseases [26,27], as well as carcinogenic activity, largely based on evidence from in vitro cell-based assays [28,29,30], making these metabolites to be largely exploitable for different purposes. 

For maximizing the recovery of bioactive compounds from plant matrixes, the Design of Experiments (DoE) represents an effective strategy. It is a statistical approach preliminary to the data collection phase, which aims to improve the knowledge and the reliability of a process. According to DoE, some parameters affecting the extraction, called factors, are varied within defined ranges, and one or more response variables are measured [31]. Several experimental designs can be used, the most common one being the Central Composite Design (CCD), which efficiently estimates first- and second-order terms, thus allowing researchers to model the response variables with curvature [32]. The DoE approach enables us to find the relationship among factors and responses, to set extraction optimal conditions and make predictions. Several studies have applied DoE for the optimization of anthocyanin extractions from grape pomace, obtaining different yields depending on type of cultivar, evaluated factors and responses, as well as the technique and solvents used [33,34,35].

In this context, the aim of this study was to select the best optimized green method for the recovery of anthocyanins from the pomace of “Sangiovese” grape vinification, the main red grape wine cultivated in Italy [36]. Two green techniques, i.e., Ultrasound- and Microwave-Assisted Extraction, coupled with three green solvents, namely acidified water, acidified 50% ethanol and a mixture of NaDES, were compared with a conventional procedure based on the stirring method with an acidified methanol/water mixture. Temperature, time, and the solid-to-liquid ratio were chosen as designed variables and their effects on two types of responses, namely the content of malvidin-3-*O*-glucoside and the antioxidant activity of the extracts, were evaluated through the DoE approach. Diverse relationships between the *X* and *Y* variables were found, and extracts with a different chemical composition were obtained under the optimal conditions, depending on the evaluated response. 

## 2. Results and Discussion

### 2.1. Comparison between Green and Conventional Extraction 

The extraction of specialized metabolites from plant material is influenced by several factors, such as the solvent type, the pH, the chemical stability of the target molecules, and specific operative parameters of the instrumentation. Temperature, time, and the solid-to-liquid ratio are among the most impacting parameters on polyphenol extraction [37,38,39]; hence, they were chosen as quantitative factors for the optimization of anthocyanin recovery from grape pomace through the DoE.

The range of values for each of these parameters was chosen based on previous research: 30–120 °C for temperature, 5–30 min for extraction time, and 0.02 to 0.07 g/mL for the solid-to-liquid ratio [34,40,41,42]. The most common anthocyanins and anthocyanidins in grape pomace, namely malvidin-3-*O*-glucoside (oenin), cyanidin-3-*O*-glucoside, petunidin-3-*O*-glucoside, peonidin-3-*O*-glucoside, malvidin, cyanidin, delphinidin-3,5-di-*O*-glucoside (delphin), cyanidin-3-*O*-galactose (ideain), cyanidin-3-*O*-rutinoside (keracyanin) and delphinidin [43,44,45,46], were searched and quantified in the extracts through HPLC-DAD analysis. 

Two green techniques, i.e., UAE and MAE, and three green solvents, i.e., acidified water, 50% acidified ethanol, and a mixture of NaDES composed of choline chloride and citric acid, were compared among each other under the central point conditions of the experimental space. Central points in DoE represent experimental runs, where the *X* variables are set halfway between the lowest and the highest values; hence, comparisons were undertaken at 75 °C, 17.5 min, 0.045 g/mL. Green methods were compared with a conventional extraction employing a non-green solvent, i.e., one typical solvent mixture commonly used for anthocyanin extraction, consisting of methanol, water and formic acid [47,48], and a non-green technique, namely the stirring method (Table 1).

Five out of ten targeted anthocyanins were found in all extracts, i.e., kuromanin, oenin, petunidin-3-*O*-glucoside, peonidin-3-*O*-glucoside, and malvidin, together with an unidentified compound not corresponding to any of the standards used. As shown in Table 1, the green methods, based on the combination of eco-friendly solvents and ultrasounds or microwaves, allowed for a better recovery of most anthocyanins compared to the conventional extraction, combining their favorable environmental impact with a good efficacy in extracting these metabolites from grape pomace matrix.

Only in the case of acidified water coupled to UAE, was the Total Anthocyanin Index (TAI), calculated as the sum of the identified anthocyanins, lower than that obtained with the conventional method (36.9 ± 0.4 mg/g DW versus 47.1 ± 0.5 mg/g DW) due to a lower yield of all individual compounds, except for malvidin. Moreover, it is worth observing that, in the case of UAE, the TAI value of the extract obtained with acidified water was about half that when using the other two solvents, while the antioxidant capacity was 3–4-fold lower (Table 1). The weak correlation between the total phenolic content and the antioxidant activity assayed by the DPPH has been observed in some cases [49], and it has been attributed to different factors, including the relative Hydrogen Atom Transfer and Single-Electron Transfer capacity of single components of the mixture, their steric accessibility to the DPPH radical, and their intrinsic interfering capacity [50]. Indeed, several compounds, such as reducing sugars, organic acids, minerals, and vitamins, which can be present in an anthocyanin extract, have been reported to influence the antioxidant capacity values based on the DPPH test [51,52]. Thus, it is possible that acidified water coupled with UAE could have extracted such interfering components to a greater extent than the ethanol/water mixture and NaDES.

Comparing the green techniques, MAE was found to be more efficient than UAE in extracting anthocyanins, and this is true for all the tested solvents (Table 1). The extraction of single metabolites was significantly improved using microwaves. The better efficiency of MAE is also reflected in the antioxidant activity of the extracts, which was higher compared to that of the extracts produced by UAE. In particular, the highest values were found in extracts obtained with ethanol/water and NaDES (29.0 and 32.4 mmol Tr Eq/g DW, respectively), which were 3-fold higher compared to those of acidified water (Table 1). Despite the lack of statistical differences between these two values, it is worth mentioning that NaDES have been recently demonstrated to exert a long-term stabilizing effect on the antioxidant capacity of extracted anthocyanins [53], thus having an added value over the ethanol/water mixture.

The different extraction capacity of MAE compared to UAE could be due to the different operating principles which these two green techniques are based on. The extraction of bioactive compounds by UAE occurs due to thermal, mechanical and cavitation effects. The thermal effect strongly depends on the solvent, which converts the ultrasonic energy into heat, and the mechanical effect is determined by the particle vibration into the medium according to mechanical waves, while the cavitation effect is due to the collapse of the microscopic bubbles forming in the medium [54]. The latter two phenomena are strictly related to ultrasound power and frequency. Thus, the efficiency of UAE depends on characteristics specifically linked to the instrumentation used, as well as on the solvent and environmental parameters [55]. Conversely, MAE is based on the direct effect of microwave energy on molecules by ionic conduction and dipole rotation, occurring simultaneously, which cause the heating of sample. When an electric field is applied, charged particles migrate through a medium, and the resistance of the solution to this flow creates friction, thus heating the solution. At the same time, by aligning with the electric field, dipolar rotation occurs, which produces molecular collisions, also resulting in heating [56]. The efficiency of microwave heating at a given frequency and temperature depends on the material and medium ability to absorb electromagnetic energy and dissipate heat. Establishing which technique is the best for anthocyanin extraction is not easy since many factors influence the final yield, thus making difficult an interlaboratory comparison. Diverse results were published concerning the comparative performance of UAE and MAE. For example, Caldas et al. [42] performed UAE using a high-intensity ultrasound processor and found a higher phenolic recovery from grape skins than that obtained with microwaves working at the same power density. Marianne et al. [57] found no significant differences in TPC and TAC in extracts obtained with MAE and UAE, even though UAE was preferred for optimization due to the shorter extraction time. Probably, in our case, the ultrasonic bath power employed during the extraction was not sufficient to provide an efficient cell matrix breakdown as much as for microwaves.

The most pronounced difference in efficiency concerned the extraction with acidified water, which performed much better with microwaves than with ultrasounds under the same conditions of temperature, solid–liquid ratio, and time (Table 1). The use of polar solvents facilitates the flow of microwaves, and this makes water one of the solvents best suited to MAE. A better performance of the acidic aqueous solution with 2 % citric acid with MAE than with UAE for grape pomace was also reported by Rocha and Noreña [58], and by Drosou et al. [59]. These authors also found a better polyphenol extraction capacity for 50% ethanol coupled with MAE than with UAE, as in our study. Interestingly, while MAE performed the best extraction with NaDES, using UAE, the highest TAI was reached with acidified 50% ethanol, even if by a small difference (75.2 ± 1.0 vs. 72.3 ± 0.7 mg/g DW), whereas for both green techniques, acidified water was found to be the least effective solvent. Based on these results, MAE was selected as the technique of choice and the extraction process was optimized for the three green solvents.

### 2.2. Optimization of MAE Extraction

MAE was optimized using a central composite face-centered design (CCF), coding each independent variable (temperature, time, and solid-to-liquid ratio) at two levels, between −1 and +1. In CCF design, the experimental region to be explored is represented by a cube, and the possible combinations of the factor levels are at the corners of this cube, while additional points are located at the center of the cube faces. The experimental model comprises 17 experiments (Table 2), eight of which are related to factorial points and are designed to capture linear and interactions effects, six experiments are related to star points and are designed to capture non-linear effects and three experiments are center points replicates [60]. Since CCF supports non-linear equations, it is generally used for response surface modeling and optimization applications [61].

Two different responses were evaluated, i.e., the antioxidant activity trough the DPPH assay and malvidin-3-*O*-glucoside (oenin) concentration since this anthocyanin was the most abundant in the extract and malvidin derivatives are among the metabolites most found in grape pomace from red grape [43,56,62]. Both responses were measured for each experimental run and the results were introduced into the design matrix. Data were treated by multiple linear regression (MLR). The fitting of the models was evaluated referring to the standard regression analysis statistical parameters.

**Figure 1:**
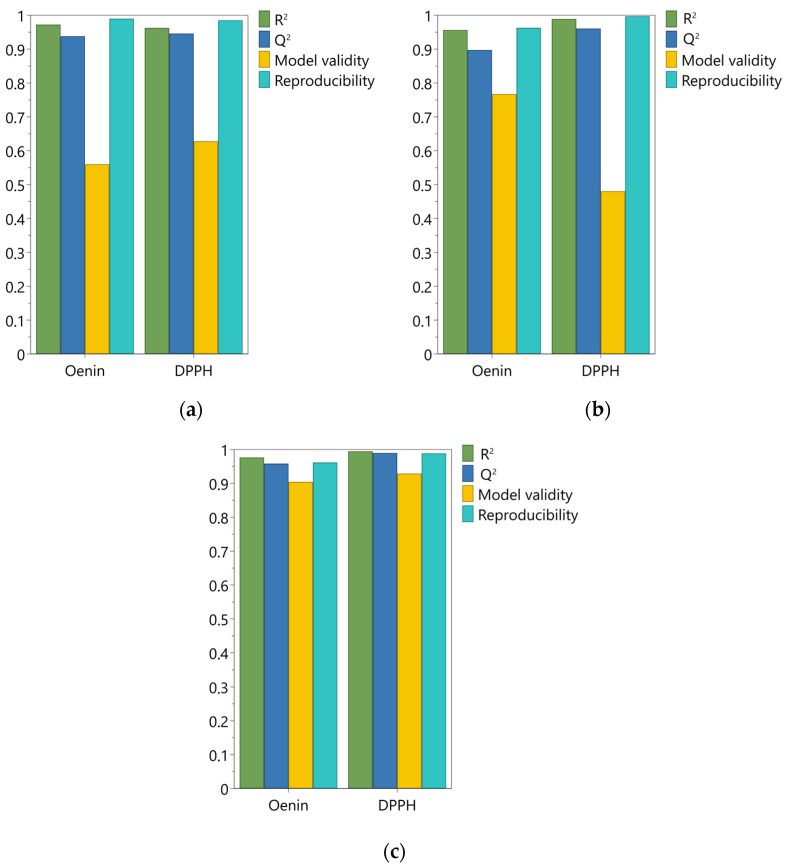
Summary of fit for the Design of Experiments models used; (**a**) 50% acidified ethanol; (**b**) acidified water; (**c**) NaDES mixture with 30% water.

As shown in Figure 1, all model summaries of fit for both responses gave good statistical parameters. The goodness of fit (R^2^), representing the variability explained by the model, ranged between 0.96 and 0.99. The goodness of prediction Q^2^ varied from 0.90 to 0.99; considering that it should be greater than 0.5 for a good model, all models had a remarkable predictive capacity. The model validity showed a wider variation, in the range of 0.48–0.93; this parameter is based on the Fisher test between the model error and the pure error, and a value higher than 0.25 means that the model has no lack of fit. Hence, albeit with some variations, all models showed a model error not significantly larger than the pure error. Finally, the reproducibility indicates the variation in the replicates compared to overall variability; as for the other parameters [63], the closer to 1 this value is, the better the model. Values for model reproducibility varied between 0.96 and 0.99 for the three solvents, indicating a great reliability for all the models (Figure 1). The ANOVA tables of the regression and residual analysis are reported in Supplementary Materials, together with the used worksheets for the development of DoE models.

#### 2.2.1. Effects of Factors on Oenin Concentration and Antioxidant Activity

**Figure 2:**
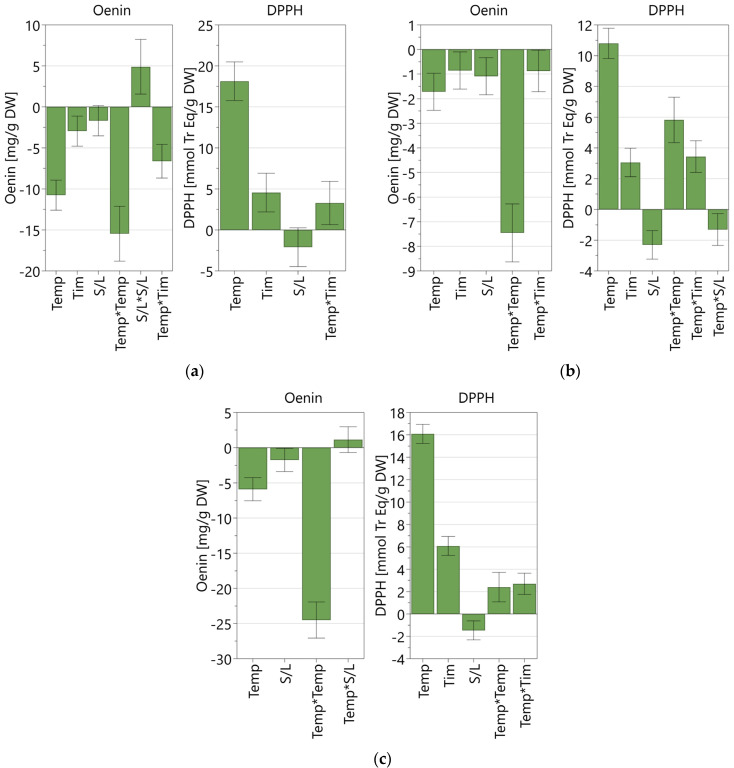
Scaled and centered coefficient plots for the three refined models obtained with MAE coupled with the three green solvents. (**a**) 50% acidified ethanol; (**b**) acidified water; (**c**) NaDES mixture with 30% water. Temp: temperature; Tim: time; S/L: solid-to-liquid ratio.

Figure 2 reports the coefficient plots for each refined DoE model. The coefficient plot provides a graphical presentation of the significance of the model terms, highlighting the influence of the studied factors over the responses. The coefficients were scaled and centered, and the error bars represent the confidence interval.

All factors considered had a statistically significant influence on both responses, showing both linear (Temp, Tim, S/L) and non-linear effects (Temp*Temp, S/L*S/L, Temp*Tim, Temp*S/L), except for the extraction time in the NaDES mixture model, which was found to have a non-significant value. For all models, the factor with the highest impact on both oenin concentration and antioxidant activity was found to be to be temperature.

The solid-to-liquid ratio had the same effect on both responses, promoting a higher antioxidant activity and oenin extraction at lower values, while the effect of temperature and time was opposite. For the antioxidant activity based on the DPPH assay, all models highlighted a positive correlation with time and temperature, while the correlation was found to be negative when considering oenin concentration measured by the HPLC-DAD analysis, as oenin extraction is reduced as time and temperature increase (Figure 2). Hence, the correlation between factors and response greatly varied depending on the response taken into consideration. 

#### 2.2.2. Process Optimization

**Figure 3:**
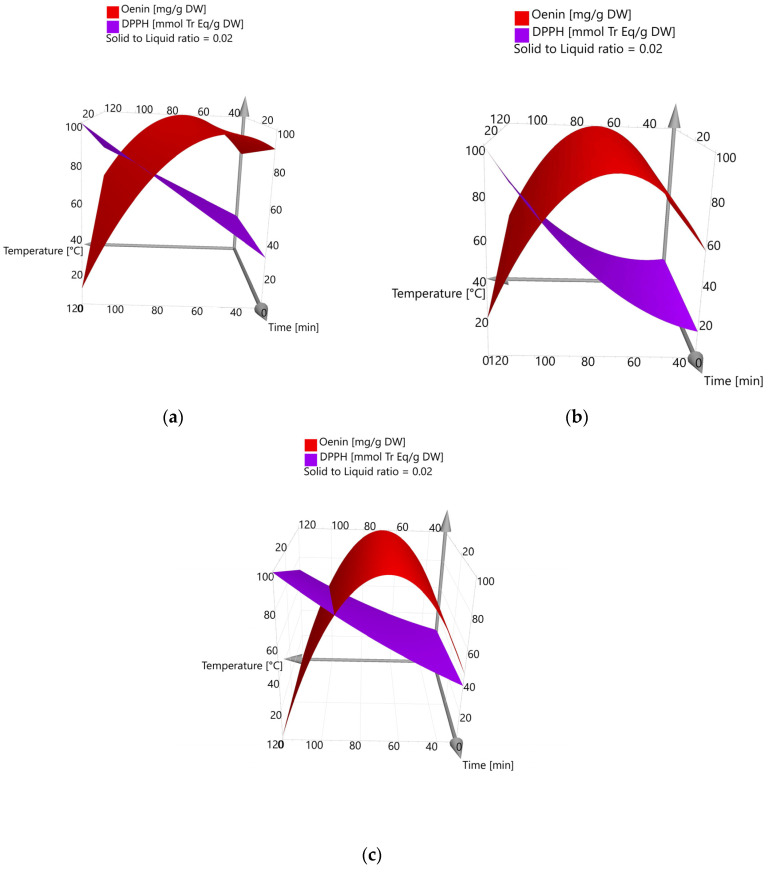
Response surface plots. (**a**) 50% ethanol acidified with 1% HCl; (**b**) water with 1% citric acid; (**c**) NaDES (choline chloride/citric acid = 2:1; 30% water).

Figure 3 shows the response surface plots for the DoE models considering both responses. These three-dimensional graphs clearly represent how the response varied as a function of all independent factors, thus allowing us to identify the optimal conditions for the best response [64]. Although with different curvatures, depending on the green solvent used, it is evident that for all DoE models, the optimal conditions fall into the selected ranges of factors for both responses. When considering oenin concentration, the experimental space was adequate to find the optimal conditions since a decrease in its recovery was observed over 80 °C due to the known anthocyanin thermolability [65]. Conversely, for antioxidant activity, the response surface suggests that higher values could be obtained by enlarging the experimental space, which means further increasing the temperature and time, over 120 °C and 30 min, respectively.

Table 3 reports the optimal extraction conditions calculated from the models for the two response variables. As concerns the antioxidant activity, the optimal extraction conditions fall at the upper extremes of time and temperature and the lowest solid-to-liquid ratio for all three solvents. The predicted antioxidant activities applying the optimal extraction conditions were 61.4, 35.8, and 60.4 mmol Tr Eq/g DW for acidified 50% ethanol, acidified water and NaDES, respectively, which were very similar to the experimental ones (Table 3). In the case of oenin concentration, the optimal extraction conditions were found at a lower temperature and extraction time, while the best solid-to-liquid ratio was again 0.02 g/mL. At optimal extraction conditions, the predicted oenin yield was 42.7 mg/g DW using acidified ethanol 50%, 13.7 mg/g DW employing acidified water and 32.3 mg/g DW with the NaDES mixture, which were confirmed experimentally. Hence, after the process optimization, the best green solvent in terms of oenin recovery was found to be 50% ethanol, which allowed for a slightly higher, but statistically significant, recovery of oenin than the NaDES mixture, while acidified water was confirmed to be the less efficient green solvent (Table 3). However, calculating the TAI as the sum of the main identified anthocyanins, no significant differences were found between the two extracts (108.1 ± 1.9 mg/g DW with 50% ethanol, 110.4 ± 0.9 mg/g DW with NaDES), since in that obtained with NaDES, the lower extraction of oenin is counterbalanced by a higher extraction of kuromanin and petunidin-3-*O*-glucoside. 

**Figure 4:**
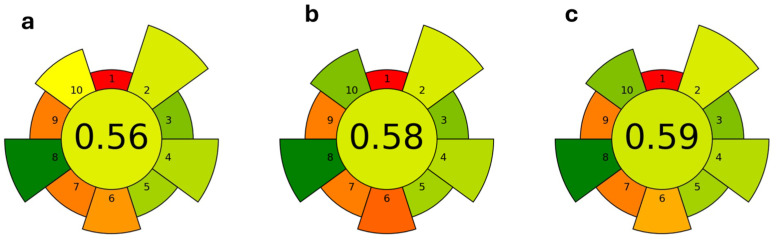
Assessment results of greenness of procedures optimized for oenin extraction from grape pomace: (**a**) 50% acidified ethanol; (**b**) acidified water; (**c**) NaDES mixture. The central, circular field corresponds to the final assessment score. It is surrounded by 10 labeled, wedge-shaped fields corresponding to each AGREEprep criterion. The color of each field, including the central field, is mapped to the particular score (0–1) through a “traffic lights” colour map (red-yellow-green), with red assigned to 0.0 and green to 1.0.

The three procedures optimized for the malvidin-3-*O*-glucoside extraction were assessed for their greenness using the AGREEprep metric tool (https://mostwiedzy.pl/pl/wojciech-wojnowski,174235-1/agreeprep; accessed on 21 May 2024), which allows us to assign a score to the sample preparation protocols by applying the 12 principles of Green Chemistry [66], and the results are shown in Figure 4. As can be seen, the scores for the three procedures based on MAE extraction were very similar to each other, with the lowest value being for the extraction with the ethanol/water mixture, and the highest for that using NaDES.

### 2.3. Critical Evaluation of DoE Approach 

**Figure 5:**
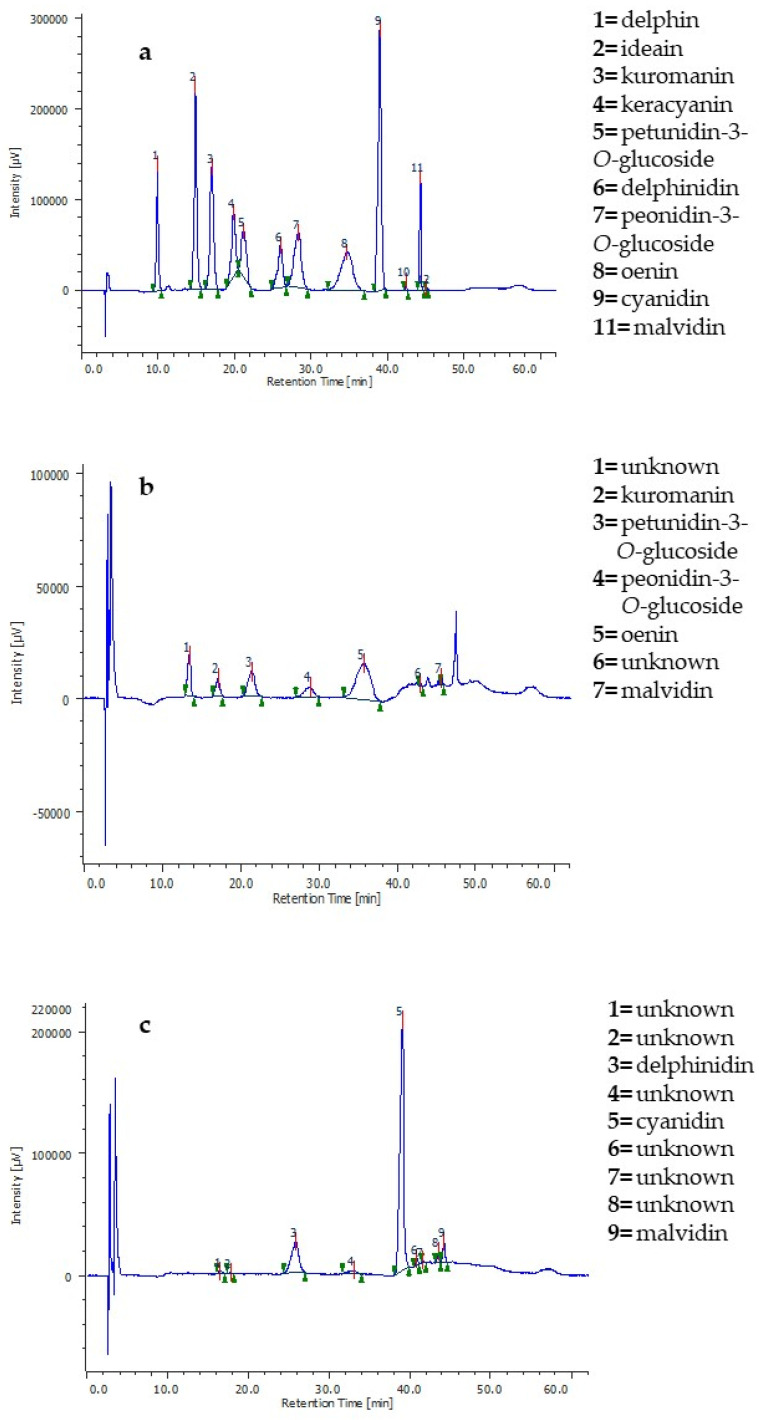
(**a**) HPLC-DAD profile of anthocyanin standards; (**b**) HPLC-DAD profile of grape pomace extract obtained with 50% ethanol optimized using the oenin content as *Y* variable; (**c**) HPLC-DAD profile of grape pomace extract obtained with 50% ethanol optimized using the antioxidant activity measured by DPPH assay as *Y* variable.

The DoE is a structured and organized statistical approach aiming to find the relationships between the factors affecting a process and the considered responses through the establishment of a mathematical model. Through this model, it is possible to achieve the optimal conditions for the best process performance and make predictions [31]. The relationships between the *X* and *Y* variables found in this study highlight that the choice of the response variable and the method for measuring it is of crucial importance since it can lead to different assessments. The extracts produced under the optimal conditions suggested by the models were analyzed by HPLC-DAD. Figure 5 shows the chromatograms of the optimized 50% ethanol extracts according to the two responses (Figure 5b,c) and that of the anthocyanin standard mix (Figure 5a).

The phytochemical profiles of the optimized extracts were very different. Making MAE at the optimal extraction conditions based on oenin concentration (i.e., 66 °C, 7.5 min, 0.02 g/mL), the extract was composed of oenin and other five main anthocyanins. Conversely, the extract produced at the optimal conditions based on the DPPH assay (i.e., 120 °C, 30 min, 0.02 g/mL) was mainly enriched in the aglycons cyanidin, delphinidin, and malvidin. Moreover, the two extracts had different colors and odors, with the latter having a brownish color and a burning smell, which are typically features associated with anthocyanin degradation [57]. Hence, the relationship found by the mathematical model considering the oenin yield can also be extended to the other anthocyanins since the chromatogram in Figure 5b confirmed that an increase in temperature and time lowers their extractive yield. This implies that the antioxidant activity of the extract produced under the optimal conditions based on the DPPH assay was not predominantly due to the molecules composing the other optimized extract since those anthocyanins were almost lost at 120 °C.

The positive correlation between temperature and antioxidant activity found by the DoE approach using the DPPH assay can be explained by the fact that under hydrothermal acidic conditions, the sugar molecules present in grape pomace undergo dehydration reactions to form furanic compounds, such as 5-hydroxymethyl-2-furfural (HMF) and derivatives [67]. These molecules can also be formed during a Maillard reaction [68] and display antioxidant activity when assayed with the most common chemical tests, thus giving rise to overestimation or interference. The DPPH of HMF was indeed previously reported to be time- and dose-dependent [69,70]. A similar interfering effect also occurs with the colorimetric assays for detecting general classes of phenolic compounds, such as Total Phenolic Content (TPC) or Total Flavonoids Content (TFC), which are very commonly used to measure the response variable in several investigations based on the optimization of phenolic extractions with a DoE approach [33,35,41,57,71]. Since acidic hydrolysis is a common practice in studying phenolics, and HMF and its derivatives can be formed under these experimental conditions, the effect of these compounds on the TPC and TFC content, as well as on the antioxidant activity measured by the DPPH assay, must be taken into consideration to avoid overestimation. Heat can cause anthocyanin degradation in a manner depending on its magnitude and the time of exposure. Moreover, the chemical structure of the single anthocyanins determines their resistance to heat; in general, inter- and intra-molecular co-pigmentation, acylation and glycosylation improve anthocyanins’ stability toward heat [72]. Therefore, different anthocyanin-containing matrices may be differently resistant to thermal degradation. For this purpose, Adams studied anthocyanin degradation under acidic aqueous conditions at 100 °C both under nitrogen and oxygen atmosphere conditions, and he found that between pH 2 and 4, the aglycon–sugar bond is the most labile; hence, the first step of thermal degradation is the hydrolysis of the sugar moiety [73]. This could explain the detection of the aglycons cyanidin, delphinidin and malvidin in the extract produced with the optimal extraction conditions determined considering the antioxidant activity measured by DPPH assay. Moreover, evidence was provided that anthocyanidins have a higher antioxidant capacity compared to anthocyanins, probably due to a lower stability of the former compared to the latter [34,74], explaining the greater antioxidant power determined for the anthocyanidin-enriched extract.

## 3. Materials and Methods

### 3.1. Plant Material

Grape pomace from *Vitis vinifera* L., “Sangiovese” cultivar, was provided by the Podere dell’Angelo winery, located in Vergiano (Rimini, Italy). Grapes used for vinification were hand-harvested between 5 and 6 September 2022. Grape pomace consisted of 81% peel, 3% stalks, 10% seeds, and 6% of residual pulp. Grape pomace was collected and immediately freeze-dried, grounded to a fine powder, and stored at −20 °C until use.

### 3.2. Chemicals

Ethanol, choline chloride, citric acid, water and acetonitrile (HPLC grade), 2,2-diphenyl-1-picrylhydrazyl, 6-hydroxy-2,5,7,8-tetramethylchroman-2-carboxylic acid were purchased from Merck Italia (Milan, Italy). Pure standards of anthocyanins (>99.5 purity) namely malvidin-3-*O*-glucoside (oenin), cyanidin-3-*O*-glucoside, petunidin-3-*O*-glucoside, peonidin-3-*O*-glucoside, malvidin, cyanidin, delphinidin-3,5-di-*O*-glucoside (delphin), cyanidin-3-*O*-galactose (ideain), cyanidin-3-*O*-rutinoside (keracyanin) and delphinidin were purchased from Extrasynthese (Lyon, France). 

### 3.3. Conventional, Microwave-, and Ultrasound-Assisted Extraction 

The conventional extract was produced using a solvent mixture composed of methanol, water and formic acid in a 60:37:3 (*v*/*v*/*v*) ratio. Briefly, 1 g of plant material was extracted with 22 mL of solvent mixture (solid-to-liquid ratio 0.045 g/mL) while stirring for one hour under nitrogen flow to prevent oxidation of anthocyanins. The extraction was repeated twice. Subsequently, the extract was filtered and evaporated under reduced pressure. 

MAE was performed using a Milestone flexiWAVE Microwave apparatus with a closed-vessel system (Milestone Srl, Sorisole, Italy). The maximum microwave power was set at 500 W; to maintain a constant temperature, it was not constantly applied. Extractions were performed at 30, 75 and 120 °C, with extraction time and the solid-to-liquid ratio varying among 5, 17.5 and 30 min and, 0.02, 0.045 and 0.07 g/mL, respectively. For each extraction, a pre-heating time of 4 min and stirring at 50% were used. The extracts were centrifugated at 2683 RCF for 5 min, and the supernatant was filtered with a 0.45 μm filter. UAE was performed with an ultrasound bath (Elmasonic S60H, Elma Schmidbauer GmbH, Singen, Germany) at the central points’ conditions (75 °C, 17.5 min, 0.045 g/mL) with a frequency of 60 kHz and heating power of 550 W. The extracts were centrifuged for 5 min at 2683 RCF, and the supernatant was filtered with a 0.45 μm filter.

### 3.4. DPPH Assay

The DPPH assay was performed according to Thaipong et al. [75] with some modifications. 12 mg of 2,2-diphenyl-1-picrylhydrazyl (DPPH) were dissolved in 50 mL ethanol to give a stock solution of 0.6 mM. Mixing 10 mL of stock solution with 45 mL of ethanol, a working solution was obtained, giving Abs of 1.1 ± 0.02 at 515 nm on a Jasco (Tokyo, Japan) V-630 double beam spectrophotometer. A calibration curve was prepared with different concentrations of 6-hydroxy-2,5,7,8-tetramethylchroman-2-carboxylic acid (Trolox) solubilized in ethanol, in the range 0.0125-1.0 mM. Samples were diluted twenty times before analysis. Then, 50 μL of ethanol (for blank measurement), Trolox (for calibration curve), or sample were added to 950 μL of the DPPH working solution. After 24 h in the dark, Absorbance was read at 515 nm. The % discoloration was calculated as follows:

% discoloration = [1 − (Abs DPPH with Trolox/Abs DPPH without Trolox)] × 100

An x,y graph showing the % discoloration as a function of Trolox concentration was used to interpolate the values obtained from sample analysis. The results were expressed as mmol Trolox Equivalent/g Dry Weight (mmol Tr Eq/g DW).

### 3.5. HPLC-DAD Analysis of Grape Pomace Extracts

HPLC-DAD analysis were carried out according to the work of Antognoni et al. [76].The extracts were injected into a Jasco (Tokyo, Japan) HPLC-DAD system consisting of a PU-4180 pump, an MD-4015 PDA detector, a CO-4061 column oven, and an AS-4050 autosampler. The stationary phase was an Agilent Zorbax Eclipse Plus C18 reverse-phase column 100 × 3 mm I.D., 3.5 μm (Santa Clara, CA, USA). The mobile phase was a mixture of 3% formic acid (*v*/*v*) (solvent A) and acetonitrile (solvent B). The initial conditions were 95% solvent A; in 3 min, the percentage of solvent A became 90% and then 89% in 27 min. Subsequently, in 5 min, solvent A’s percentage became 80%, and after 5 min, 70%. In the following 5 min, the percentage of solvent A was restored to 95%, which was kept constant for 13 min. The flow rate was 0.4 mL/min, the oven temperature was set at 35 °C, the injection volume was 20 μL for all determinations, and analyte detection was carried out with a diode array detector (DAD) by monitoring at 530 nm. Quantification was performed with pure standards using calibration curves ranging between 80 and 0.0625 μg mL^−1^ (R^2^ ≥ 0.9861). Extracts from UAE and MAE were directly injected after extraction, and the conventional extract was analyzed by injecting it at a concentration of 5 mg/mL. 

### 3.6. Design of Experiments (DOE)

A full factorial Central Composite Face design (CCF) was employed, analyzing the factors over three levels of variations, namely 120, 75, and 30 °C for temperature, 5, 17.5 and 30 min for the extraction time, and 0.02, 0.045 and 0.07 g/mL for the solid-to-liquid ratio. As shown in Table 2, 17 planned experiments were set, 3 of which (N15, N16 and N17) consisted of center points. The experimental runs were performed in a randomized order to reduce the risk of systematic errors. Two types of responses, namely oenin content through HPLC-DAD analysis, and antioxidant activity through DPPH assay, were evaluated. MODDE^®^ Pro 13.0.2 software (Sartorius Stedim Biotech, Göttingen, Germany) was employed to develop the DoE models, permitting us to study the experimental variability, the effect of the process variables, and assessing the optimal extraction conditions.

### 3.7. Statistical Analyses

Statistical analyses were carried out with R software version 4.2.2 (2022-10-31).

Samples were compared by one-way analysis of variance (ANOVA) using “aov” function from “stats” package. Tukey’s Honest Difference (HSD) post hoc test was carried out using Tukey HSD function considering a significant difference at *p* values < 0.05. The assessment score for the greenness of the optimized procedures was performed according to the AGREEprep, as described by Wojnowski et al. (2022) [66].

## 4. Conclusions

Grape pomace is a by-product still rich in valuable anthocyanins that can be efficiently recovered with a sustainable approach combining green methods and green solvents instead of the highly environment-impacting conventional procedures. Microwaves were found to be more efficient than ultrasounds, and for both techniques, 50% ethanol and the NaDES mixture yielded higher anthocyanin recovery compared to acidified water. The optimization of MAE by the DoE approach showed that the method for recording the investigated response must be carefully chosen to ensure the production of an extract with a phytochemical profile as close as possible to the original matrix. The results indicated that when increasing the temperature over a certain threshold, degradation phenomena occur, leading to the production of an anthocyanidin-enriched extract; however, by maintaining the temperature below that value, an anthocyanin-enriched extract was obtained. This should be evaluated depending on the final application of the optimized extract; in case of a pharmaceutical or supplement application, it is important to maintain a well-defined chemical composition of the extract, and this makes a more accurate analysis, such as HPLC-DAD, a better tool for measuring anthocyanin recovery.

The feasibility of applying MAE for recovering bioactive compounds at an industrial scale from several matrices has been investigated by several authors [77,78] taking into account safety considerations. Regarding the grape pomace used in this study, its main composition in grape skin makes this matrix potentially exploitable by the food, cosmetic, and supplement industries to obtain food additives, antimicrobial compounds, fiber, and functional ingredients. Moreover, an anthocyanin-enriched extract could also be exploited by the agrochemical industries to obtain fertilizers or biostimulants given the well-recognized capacity of phenolic compounds to affect plant growth and plants’ response to abiotic stresses [79]. Experiments are in progress to investigate the application of the three optimized anthocyanin-enriched extracts from grape pomace as potential biostimulants for improving the response of model plants to abiotic stresses through a seed-priming approach.

## Figures and Tables

**Table 1:** Comparison of UAE, MAE and conventional extraction of anthocyanins from grape pomace. Anthocyanin concentrations and total anthocyanin index (TAI) were expressed as mg/g DW; antioxidant activity was expressed as mmol Trolox Equivalent/g DW. Superscript letters within the same column refer to statistical analysis, with different letters indicating significant differences (*p* ≤ 0.05) in the metabolite content or in antioxidant activity.

UAE	Kuromanin	Oenin	Pet-3- *O*-glu *	Peo-3- *O*-glu *	Malvidin	TAI	A.A. *
acidified EtOH 50%	5.0 ± 0.5 ^a^	22.2 ± 1.8 ^c^	19.1 ± 1.2 ^b^	12.9 ± 1.3 ^bc^	15.9 ± 0.8 ^ab^	75.2 ± 1.0 ^bc^	20.4 ± 1.2 ^c^
acidified H_2_O	2.4 ± 0.1 ^c^	7.8 ± 0.7 ^d^	7.6 ± 0.1 ^d^	5.7 ± 0.2 ^e^	13.3 ± 0.2 ^a^	36.9 ± 0.4 ^e^	5.1 ± 0.2 ^b^
NaDES	4.6 ± 0.4 ^a^	23.1 ± 0.7 ^c^	18.5 ± 0.4 ^b^	11.4 ± 0.8 ^cd^	14.7 ± 0.6 ^ab^	72.3 ± 0.7 ^cd^	17.4 ± 1.4 ^c^
**MAE**							
acidified EtOH 50%	6 ± 0.7 ^ab^	29.5 ± 0.7 ^a^	20.7 ± 1.6 ^ab^	15.5 ± 1.2 ^ab^	14.8 ± 0.9 ^ab^	86.1 ± 2.3 ^ab^	29 ± 2.0 ^a^
acidified H_2_O	4.9 ± 0.3 ^a^	13.0 ± 1.5 ^b^	12.2 ± 0.2 ^c^	10.3 ± 0.7 ^cd^	13.9 ± 0.3 ^a^	54.4 ± 1.1 ^cd^	10.0 ± 0.2 ^b^
NaDES	8.2 ± 0.2 ^b^	32.5 ± 2.6 ^a^	24.4 ± 2.1 ^a^	17.3 ± 0.6 ^a^	17.2 ± 2.4 ^b^	99.6 ± 2.5 ^a^	32.4 ± 2.3 ^a^
**Conventional**							
MeOH/H_2_O/Formic acid 60/37/3 (*v*/*v*/*v*)	3.4 ± 0.02 ^ac^	18.4 ± 0.5 ^e^	13.3 ± 0.4 ^c^	7.5 ± 0.1 ^de^	3.7 ± 0.2 ^c^	47.1 ± 0.5 ^de^	6.2 ± 0.4 ^b^

* Pet-3-*O*-glu: Petunidin-3-*O*-glucoside; Peo-3-*O*-glu: Peonidin-3-*O*-glucoside; A.A.: Antioxidant activity.

**Table 2:** Worksheet set by DoE for optimization of anthocyanins’ extraction from grape pomace.

Exp No	Exp Name	Run Order	Temperature [°C]	Time [min]	Solid-to-Liquid Ratio [g/mL]
1	N1	3	30	5	0.02
2	N2	6	120	5	0.02
3	N3	11	30	30	0.02
4	N4	10	120	30	0.02
5	N5	8	30	5	0.07
6	N6	4	120	5	0.07
7	N7	7	30	30	0.07
8	N8	16	120	30	0.07
9	N9	17	30	17.5	0.045
10	N10	9	120	17.5	0.045
11	N11	13	75	5	0.045
12	N12	12	75	30	0.045
13	N13	14	75	17.5	0.02
14	N14	15	75	17.5	0.07
15	N15	2	75	17.5	0.045
16	N16	1	75	17.5	0.045
17	N17	5	75	17.5	0.045

**Table 3:** Optimal conditions calculated from the RSM models, predicted and experimental values for each response. Superscript letters within the same column refer to statistical analysis; different letters indicate significant differences (*p* < 0.05).

Green Solvent	A.A. * [mmol Tr E/g DW]	Predicted vs. Experimental	Oenin [mg/g DW]	Predicted vs. Experimental
50% Ethanol acidified with 1%HCl	120 °C 30 min 0.02 g/mL	61.4 vs. 61.0 ± 1.2 ^a^	66 °C 7.5 min 0.02 g/mL	42.7 vs. 41.2 ± 2.0 ^a^
Water acidified with 1% citric acid	120 °C 30 min 0.02 g/mL	35.8 vs. 35.7 ± 0.7 ^b^	75 °C 17.5 min 0.02 g/mL	13.7 vs. 15.5 ± 0.8 ^b^
NaDES (choline chloride/citric acid = 2:1; 30% water)	120 °C 30 min 0.02 g/mL	60.4 vs. 60.8 ± 1.9 ^a^	75 °C 5 min 0.02 g/mL	32.3 vs. 35.1 ± 1.3 ^c^

* A.A.: antioxidant activity.

## Data Availability

Data presented in this study are available upon request.

## Supplementary Materials

The following supporting information can be downloaded at: https://www.mdpi.com/article/10.3390/molecules29112679/s1, Table S1: ANOVA table for a complete summary of the regression and residual analysis for ethanol 50% using oenin content or antioxidant activity as Y variables; Table S2: ANOVA table for a complete summary of the regression and residual analysis for acidified water using oenin content or antioxidant activity as Y variables; Table S3: ANOVA table for a complete summary of the regression and residual analysis for NaDES mixture (choline chloride/citric acid = 2:1, 30% water) using oenin content or antioxidant activity as Y variables; Table S4: Worksheet for the development of DoE models using ethanol 50% as green solvent; Table S5: Worksheet for the development of DoE models using acidified water as green solvent; Table S6: Worksheet for the development of DoE models using NaDES mixture (choline chloride/citric acid = 2:1, 30% water) as green solvent.

## Abbreviations

DoE	Design of Experiments
DPPH	2,2-Diphenyl-1-picrylhydrazyl
DW	Dry Weight
HMF	5-hydroxymethyl-2-furfural
HPLC-DAD	High-Performance Liquid Chromatography with Diode Array Detection
MAE	Microwave-Assisted Extraction
RSM	Response Surface Methodology
TAC	Total Anthocyanin Content
TAI	Total Anthocyanin Index
TFC	Total Flavonoid Content
TPC	Total Phenolic Content
UAE	Ultrasound-Assisted Extraction
