# Supplementary material for: Green Method Comparison and Optimization of Anthocyanin Recovery from “Sangiovese” Grape Pomace: A Critical Evaluation of the Design of Experiments Approach
Source: Molecules. 2024 Jun 5;29(11):2679. doi: 10.3390/molecules29112679 (PMC11173428; doi:10.3390/molecules29112679)
Supplement: Supplementary file 1 [file molecules-29-02679-s001.zip › molecules-3017290-supplementary.pdf]

Supplementary Materials

Table S1: ANOVA table for a complete summary of the regression and residual analysis for ethanol 50% using as Y variables oenin content or antioxidant activity.

**Oenin**	**DF**	**SS**	**MS (variance)**	**F**	**p**	**SD**
Total	17	58.5062	3.44154			
Constant	1	58.4304	58.4304			
Total corrected	16	0.0757764	0.00473603			0.0688188
Regression	6	0.0699075	0.0116513	19.8525	**0.000**	0.107941
Residual	10	0.00586892	0.000586892			0.0242259
Lack of fit (Model error)	8	0.00574557	0.000718196	11.6443	**0.081**	0.0267992
Pure error	2	0.000123356	6.1678e-05			0.00785354
(Replicate error)	N = 17	Q2 = 0.788	Cond. No = 3.978			
	DF = 10	R2 = 0.923	RSD = 0.02423			
		R2 adj. = 0.876				
**DPPH**	**DF**	**SS**	**MS (variance)**	**F**	**p**	**SD**
Total	17	22617.5	1330.44			
Constant	1	18852.4	18852.4			
Total corrected	16	3765.14	235.321			15.3402
Regression	4	3624.28	906.07	77.1908	**0.000**	30.101
Residual	12	140.857	11.7381			3.42609
Lack of fit (Model error)	10	133.81	13.381	3.79774	**0.226**	3.658
Pure error	2	7.04683	3.52342			1.87708
(Replicate error)	N = 17	Q2 = 0.946	Cond. No = 1.458			
	DF = 12	R2 = 0.963	RSD = 3.426			
		R2 adj. = 0.950				

Table S2: ANOVA table for a complete summary of the regression and residual analysis for acidified water using as Y variables oenin content or antioxidant activity.

**Oenin**	**DF**	**SS**	**MS (variance)**	**F**	**p**	**SD**
Total	17	2794.07	3.44154			
Constant	1	2071.34	2071.34			
Total corrected	16	722.728	45.1705			6.7209
Regression	6	637.395	106.232	12.4491	**0.000**	10.3069
Residual	10	85.3335	8.53335			2.92119
Lack of fit (Model error)	8	80.5397	10.0675	4.20023	**0.206**	3.17293
Pure error	2	4.79377	2.39688			1.54819
(Replicate error)	N = 17	Q2 = 0.756	Cond. No = 3.978			
	DF = 10	R2 = 0.882	RSD = 2.921			
		R2 adj. = 0.811				
**DPPH**	**DF**	**SS**	**MS (variance)**	**F**	**p**	**SD**
Total	17	2692.2	158.365			
Constant	1	1983.89	1983.89			
Total corrected	16	708.314	44.2696			6.65354
Regression	4	678.564	169.641	68.4272	**0.000**	13.0246
Residual	12	29.7498	2.47915			1.57453
Lack of fit (Model error)	10	29.1838	2.91838	10.3132	**0.092**	1.70833
Pure error	2	0.56595	0.282975			0.531954
(Replicate error)	N = 17	Q2 = 0.931	Cond. No = 2.88			
	DF = 12	R2 = 0.958	RSD = 1.575			
		R2 adj. = 0.944				

Table S3: ANOVA table for a complete summary of the regression and residual analysis for NaDES mixture (choline chloride: citric acid= 2:1, 30% water) using as Y variables oenin content or antioxidant activity.

**Oenin**	**DF**	**SS**	**MS (variance)**	**F**	**p**	**SD**
Total	17	21674.9	1274.99			
Constant	1	18599.1	18599.1			
Total corrected	16	3075.75	192.234			13.8649
Regression	6	3059.36	611.871	410.593	**0.000**	24.736
Residual	10	16.3923	1.49021			1.22074
Lack of fit (Model error)	8	12.0135	1.33483	0.60967	**0.753**	1.15535
Pure error	2	4.37887	2.18943			1.47967
(Replicate error)	N = 17	Q2 = 0.990	Cond. No = 2.88			
	DF = 11	R2 = 0.995	RSD = 1.221			
		R2 adj. = 0.992				
**DPPH**	**DF**	**SS**	**MS (variance)**	**F**	**p**	**SD**
Total	17	7309.41	429.965			
Constant	1	4290.4	4290.4			
Total corrected	16	3019	188.688			13.7364
Regression	4	2897.21	965.737	103.081	**0.000**	31.0763
Residual	12	121.794	9.36874			3.06084
Lack of fit (Model error)	10	107.773	9.79751	1.39756	**0.490**	3.1301
Pure error	2	14.0209	7.01046			2.64773
(Replicate error)	N = 17	Q2 = 0.939	Cond. No = 2.88			
	DF = 13	R2 = 0.960	RSD = 3.061			
		R2 adj. = 0.950				

Table S4: Worksheet for the development of DoE models using ethanol 50% as green solvent.

Exp No	Exp Name	Run Order	Temperature [°C]	Time [min]	Solid-to-liquid ratio [g/mL]	Oenin	DPPH
1	N1	3	30	5	0.02	31.0509	14.3498
2	N2	6	120	5	0.02	23.6749	45.035
3	N3	11	30	30	0.02	38.6589	17.3638
4	N4	10	120	30	0.02	2.22592	60.9664
5	N5	8	30	5	0.07	27.5725	12.5003
6	N6	4	120	5	0.07	19.5637	41.6237
7	N7	7	30	30	0.07	33.8572	13.077
8	N8	16	120	30	0.07	1.9852	55.5651
9	N9	17	30	17.5	0.045	31.3657	19.8575
10	N10	9	120	17.5	0.045	7.41471	55.2648
11	N11	13	75	5	0.045	38.3363	29.0773
12	N12	12	75	30	0.045	33.9343	41.0761
13	N13	14	75	17.5	0.02	41.8573	39.6097
14	N14	15	75	17.5	0.07	37.6113	33.5711
15	N15	2	75	17.5	0.045	29.792	28.0949
16	N16	1	75	17.5	0.045	29.4715	27.8624
17	N17	5	75	17.5	0.045	31.7848	31.2236

Table S5: Worksheet for the development of DoE models using acidified water as green solvent.

Exp No	Exp Name	Run Order	Temperature [°C]	Time [min]	Solid-to-liquid ratio [g/mL]	Oenin	DPPH
1	N1	14	30	5	0.02	7.96454	5.99366
2	N2	8	120	5	0.02	5.8979	23.935
3	N3	6	30	30	0.02	8.35633	5.3873
4	N4	11	120	30	0.02	1.78445	35.6825
5	N5	9	30	5	0.07	6.25221	3.43866
6	N6	10	120	5	0.07	4.95318	14.7622
7	N7	5	30	30	0.07	5.21813	3.31426
8	N8	12	120	30	0.07	1.41991	29.7521
9	N9	2	30	17.5	0.045	6.85569	4.08155
10	N10	1	120	17.5	0.045	3.38421	17.3915
11	N11	16	75	5	0.045	11.596	5.36828
12	N12	13	75	30	0.045	11.3086	9.88747
13	N13	15	75	17.5	0.02	15.5474	11.6516
14	N14	17	75	17.5	0.07	10.8326	8.34375
15	N15	7	75	17.5	0.045	13.8637	9.76094
16	N16	4	75	17.5	0.045	12.2282	10.0934
17	N17	3	75	17.5	0.045	13.2581	10.8024

Table S6: Worksheet for the development of DoE models using NaDES mixture (choline chloride: citric acid= 2:1, 30% water) as green solvent.

Exp No	Exp Name	Run Order	Temperature [°C]	Time [min]	Solid-to-liquid ratio [g/mL]	Oenin	DPPH
1	N1	4	30	5	0.02	13.2788	15.2365
2	N2	14	120	5	0.02	0.481575	43.2659
3	N3	16	30	30	0.02	16.4539	22.11
4	N4	13	120	30	0.02	0	60.8524
5	N5	15	30	5	0.07	8.55195	12.6726
6	N6	10	120	5	0.07	0.165343	39.7611
7	N7	12	30	30	0.07	11.8545	19.2147
8	N8	7	120	30	0.07	0	57.1426
9	N9	3	30	17.5	0.045	9.46954	20.6641
10	N10	5	120	17.5	0.045	0	49.714
11	N11	6	75	5	0.045	32.6233	25.1498
12	N12	17	75	30	0.045	26.1453	37.5214
13	N13	8	75	17.5	0.02	34.8704	32.1479
14	N14	1	75	17.5	0.07	27.0919	30.1111
15	N15	9	75	17.5	0.045	32.2158	30.7114
16	N16	11	75	17.5	0.045	32.8417	32.3643
17	N17	2	75	17.5	0.045	27.9749	33.6637
